# Supplementary figures and images for: ViralRecall—A Flexible Command-Line Tool for the Detection of Giant Virus Signatures in ‘Omic Data
Source: Viruses. 2021 Jan 20;13(2):150. doi: 10.3390/v13020150 (PMC7909515; doi:10.3390/v13020150)

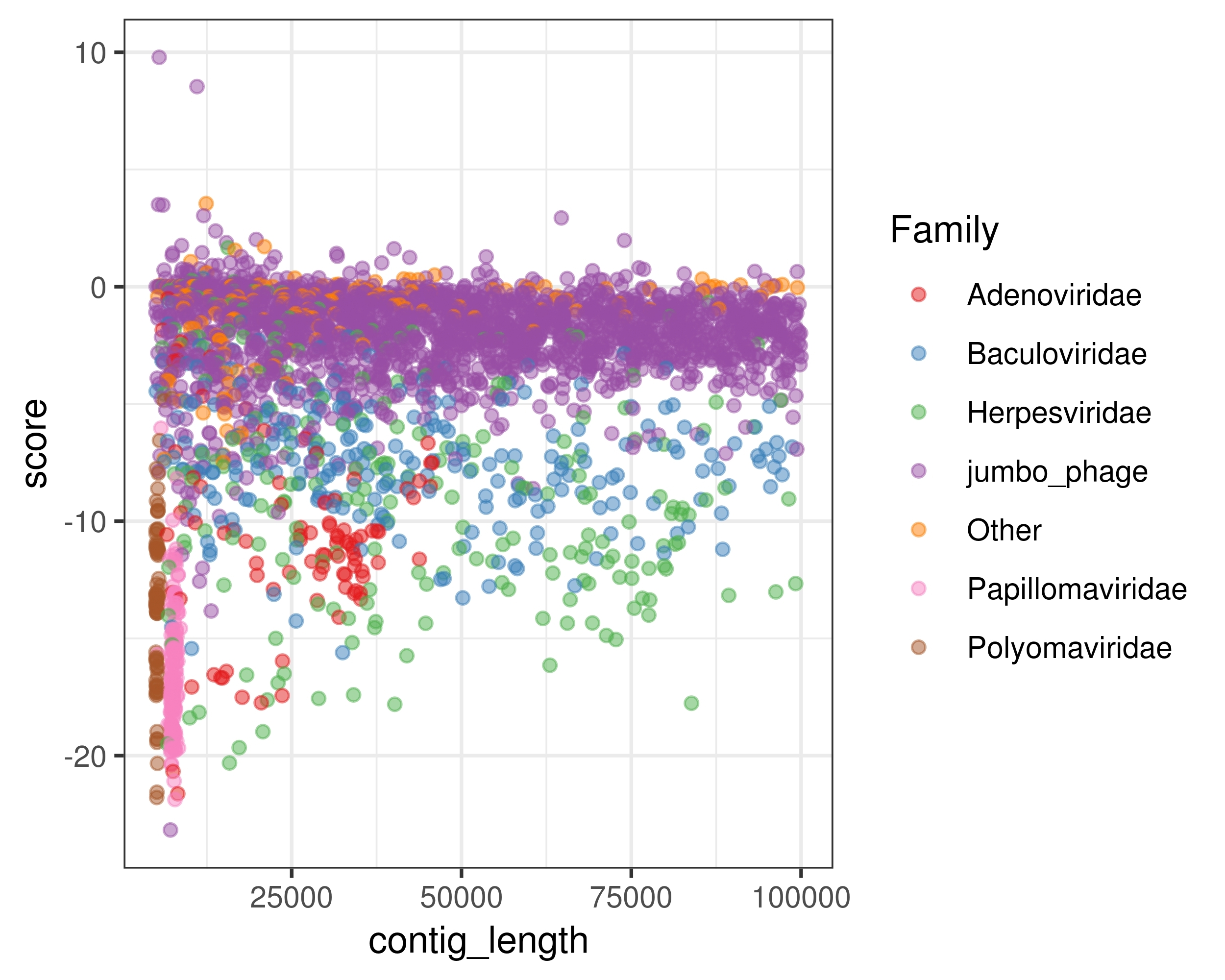

Supplement: Supplementary file 1 [file viruses-13-00150-s001.zip › suppl/Figure_S1.jpg]
